# Association of resilience and psychological flexibility with surgeons’ mental wellbeing

**DOI:** 10.1093/bjsopen/zrae060

**Published:** 2024-07-23

**Authors:** Maddy Greville-Harris, Catherine Withers, Agata Wezyk, Kevin Thomas, Helen Bolderston, Amy Kane, Sine McDougall, Kevin J Turner

**Affiliations:** Department of Psychology, Poole House, Bournemouth University, Poole, UK; Department of Psychology, Poole House, Bournemouth University, Poole, UK; Department of Psychology, Poole House, Bournemouth University, Poole, UK; Department of Psychology, Poole House, Bournemouth University, Poole, UK; Department of Psychology, Poole House, Bournemouth University, Poole, UK; Department of Psychology, Poole House, Bournemouth University, Poole, UK; Department of Psychology, Poole House, Bournemouth University, Poole, UK; Department of Psychology, Poole House, Bournemouth University, Poole, UK; Department of Urology, University Hospitals Dorset, Bournemouth, UK

## Abstract

**Background:**

Existing research highlights the link between certain personality traits and mental health in surgeons. However, little research has explored the important role of psychological skills and qualities in potentially explaining this link. A cross-sectional survey of UK-based surgeons was used to examine whether two such skills (psychological flexibility and resilience) helped to explain why certain personality traits might be linked to mental health in surgeons.

**Method:**

An online survey comprising measures of personality (neuroticism, extraversion and conscientiousness), psychological skills/qualities (psychological flexibility and resilience) and mental health (depression, anxiety, stress and burnout) was sent to surgeons practising in the UK. Mediation analyses were used to examine the potential mediating role of psychological flexibility and resilience in explaining the relationship between personality factors and mental health.

**Results:**

A total of 348 surgeons completed the survey. In all 12 mediation models, psychological flexibility and/or resilience played a significant role in explaining the relationship between personality traits (neuroticism, extraversion and conscientiousness) and mental health (depression, anxiety and burnout).

**Conclusion:**

Findings suggest that it is not only a surgeon’s personality that is associated with their mental health, but the extent to which a surgeon demonstrates specific psychological qualities and skills (psychological flexibility and resilience). This has important implications for improving surgeons’ mental wellbeing, because psychological flexibility and resilience are malleable, and can be successfully targeted with interventions in a way that personality traits cannot.

## Teaser

This study examined whether two malleable psychological skills (psychological flexibility and resilience) mediated the relationship between personality traits and mental health in surgeons. Our findings suggest that it is not only a surgeon’s personality that predicts their mental health, but the extent to which a surgeon demonstrates these specific psychological skills. This has important implications for improving surgeons’ mental wellbeing, because psychological skills can be successfully targeted with interventions in a way that personality traits cannot.

## Introduction

Over 50% of surgeons are estimated to experience burnout (physical, emotional and mental exhaustion resulting from the demands of their work), which is increasing at an alarming rate^1^. Shanafelt *et al*^2^. found that burnout rates were higher among US surgeons relative to other employees within and beyond the medical profession. Among surgeons, burnout is linked to detrimental outcomes, including higher rates of alcohol misuse^3^, medical errors^1^ and suicidal ideation^4^.

Likely related to the demands of their work, many surgeons experience mental health difficulties. High rates of depression and anxiety have been reported in surgeons^5^ compared with other non-surgical medical professionals^6^. Sadly, levels of suicidal ideation are estimated to be 1.5–3 times higher among surgeons than the general population^4^.

Adverse events in patients also impact surgeons’ wellbeing, leading to increased burnout, stress, depression and anxiety^7^. In a UK study^8^, over 35% of surgeons (*n* = 445) reported clinically significant levels of posttraumatic stress symptoms after experiencing an adverse surgical event, with nearly half reporting symptoms of anxiety.

While many such difficulties are undoubtedly related to work conditions, inherent characteristics of the surgeon’s personality will inevitably play a role. Surgeons typically show a particular personality profile: low levels of neuroticism (the predisposition to experience greater levels of negative emotion and perceptions of threat)^9^, high levels of conscientiousness (characterized by diligence, discipline and motivation to reach goals)^10^ and high extraversion (characterized by being cheerful, sociable and energetic)^11^, compared with the general population^12^.

These personality characteristics are important in predicting mental health, serving as protective/risk factors for later difficulties^13^. Higher conscientiousness and extraversion, and lower neuroticism predict better mental wellbeing^14^. Higher neuroticism has been linked to greater emotional exhaustion in healthcare staff^15^, medical students^16^ and neurosurgeons^17^, and increased risk of burnout in medical residents^18^. In contrast, higher extraversion and conscientiousness predict better mental health, lower stress and lower burnout in medical professionals^19–25^. Such research highlights the important role of personality traits in relation to mental health for surgeons.

While recognizing that some personality traits may increase the risk of poorer mental health for surgeons, key reasons for exploring psychological factors in the relationship between personality and mental health are that: personality, while arguably not totally fixed is not easily amenable to change, and excluding all surgeons with certain personality traits from the workforce would be neither practical nor desirable^12^. Thus, a more useful approach could be to target the surgeon’s capacity to withstand the rigours of surgical life rather than focusing on intrinsic personality traits. Two candidate psychological factors are resilience and psychological flexibility (PF).

Resilience is defined as the ability to bounce back^26^ to ‘harness resources to sustain wellbeing’^27^, ‘recover rapidly from difficult situations ...(and) endure ongoing hardship’^28^. Resilience is a learnable skill and can be targeted with interventions for healthcare workers^29^. Resilience among surgeons can serve as a protective factor for mental wellbeing and has been linked to improved patient care^30^.

Similarly, PF plays a role in quality of life and burnout in healthcare professionals^31^. PF involves noticing and accepting the present moment, and acting in alignment with personal values, despite the presence of any challenging or distressing thoughts, emotions, memories or physical sensations^32^. Like resilience, PF is a malleable factor and is a key target for psychological intervention^33,34^. Promisingly, high PF is associated with better quality of life in healthcare professionals^35^ although very little research has focused specifically on surgeons^36,37^.

While there is evidence linking personality traits and mental health in surgeons^17^, little is understood about how these traits interact with a surgeon’s psychological resources (that is resilience and PF). The mediating role of PF in the relationship between personality factors and mental health has been examined in the general population^38^ and medical students^39^, but not among surgeons.

Determining whether such a relationship exists is vital in helping to prevent and improve poor mental health among surgeons, as both PF and resilience, unlike personality traits, can be enhanced with tailored interventions^40,41^. Moreover, understanding the relationship between psychological factors (resilience and PF), personality traits and mental health can help tailor psychological interventions to surgeons displaying specific personality traits.

This work investigates whether resilience and PF mediate (explain) the relationship between key personality traits (neuroticism, extraversion and conscientiousness) and mental health (depression, stress, anxiety and burnout) in UK surgeons.

## Methods

### Participants and recruitment

Opportunistic sampling was used to recruit participants through advertisement via oral presentations and word of mouth at surgeon events (for example oral presentation at the annual meeting of the British Association of Urological Surgeons) and through the Royal College of Surgeons of England (RCS) adverts and e-mail distribution. Practising UK-based surgeons were eligible to participate in an online survey hosted on Qualtrics XM^42^. Given the lack of existing mediation research in this area, it was difficult to estimate an appropriate sample size. However, Fritz and MacKinnon’s guidelines^43^ suggest that for mediation analyses with a power of 0.8, a sample of 558 is needed to detect very small effect sizes, with only 162 participants needed to detect small-medium effects. The authors therefore aimed for a pragmatic sample size between these two estimates (approximately *n* = 360).

### Measures

#### Demographics

Participants were asked demographic questions before completing the questionnaires (age, sex, specialty, grade—that is whether consultant or training grade, and if training grade, what stage of training).

#### Personality dimensions – neuroticism, extraversion and conscientiousness

The Big Five Inventory (BFI) is a 44-item scale to assess the five personality dimensions of openness, conscientiousness, neuroticism, extraversion and agreeableness^44^. This inventory is widely used, with good psychometric properties and high internal consistency^45^. The authors utilized three subscales: neuroticism (N) (8 items), extraversion (E) (8 items) and conscientiousness (C) (9 items).

#### Psychological flexibility (PF)

The Work-Related Acceptance and Action Questionnaire (WAAQ) measures PF in work environments^46^. This seven-item scale is a valid and reliable measure of PF^47^.

#### Resilience

The Brief Resilience Scale (BRS) is a measure of resilience assessing the ability to bounce back and recover from stress^47^. This six-item scale has good psychometric properties when compared with other resilience measures^48^.

#### Mental health status – depression, anxiety and stress

The Depression Anxiety and Stress Scale-Short Form (DASS-21)^49^ is a widely used measure of depression (D), anxiety (A) and stress (S)^50^. Each was measured on a seven-item subscale. As recommended, subscale and total scores were doubled to give scores consistent with the full DASS-42 scale^49^.

#### Burnout

The Copenhagen Burnout Inventory (CBI) is a measure of burnout, focusing specifically on physical and psychological fatigue and exhaustion^51^. This 19-item inventory has good psychometric properties^52^ and measures burnout across personal (P), work-related (WR) and client-related (CR) domains with three subscales. For brevity, given that the total score has been used in previous research with surgeons^53^, the authors used the total CBI score in their mediation analyses.

### Procedure

Ethical approval was obtained from the Bournemouth University Ethics panel (ID: 12613). Potential participants were initially directed to an online information sheet and consent statements. Consenting participants were then asked to complete the battery of questionnaires (above) before being directed to a debrief. Survey items were not mandatory. Anonymized survey responses were analysed using Statistical Package for the Social Sciences (SPSS, IBM Statistics, version 26).

### Data analysis plan

A small amount of data was missing in the data set (less than 1.5% missing on scale measures). Participants who missed more than one item on a subscale had their subscale responses removed (*n* = 7). The minimal remaining missing data was inputted using the widely used expectation maximization method^54^.

**Fig. 1:**
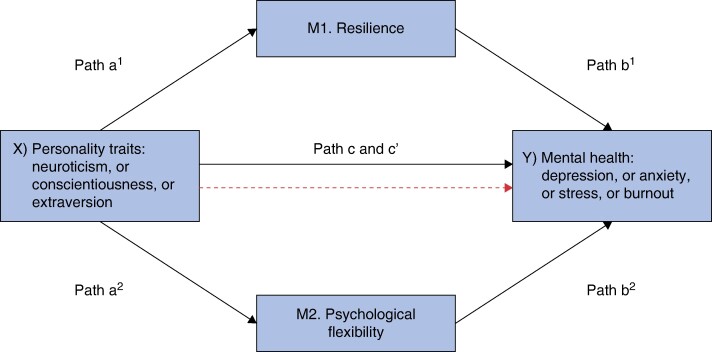
Illustration of the mediation models

Mediation analyses explored whether resilience and PF acted as mediators in the relationship between personality traits and mental health; that is, whether resilience and PF played a significant role in those relationships. Mediation analyses were performed using the PROCESS macro tool for SPSS—an established method for detecting mediation effects^55^. Using bootstrapping (with the number of samples set to 5000), the authors used PROCESS Model 4 to examine whether personality factors (X) influenced mental health (Y) via the mediators of resilience (M1) and PF (M2). Thus, they ran 12 mediation models in total (see *Fig. 1* for an illustration), to examine the proposed mediation model with each personality factor (neuroticism, conscientiousness, extraversion) for each mental health measure (depression, anxiety, stress and burnout). Given the lack of consensus for power analysis for mediation models with multiple mediators, the authors aimed for a sample of approximately *n* = 360 in line with estimates from Fritz and MacKinnon^43^. The authors followed Hayes pragmatic guidelines to optimize statistical power by collecting the largest sample size possible within their resource constraints^55^. Effect sizes were calculated using the completely standardized indirect effect.

Twelve mediation models were run using this format (M = mediator, X = predictor and Y = outcome variable). Both mediators (resilience and psychological flexibility) were entered into each model. The models tested the mediated relationship between each personality trait (neuroticism, conscientiousness, extraversion) and each mental health measure (depression, anxiety, stress, burnout) separately. The black arrow represents the direct pathway, and the red arrow represents the indirect pathway.

## Results

After the removal of two participants above retirement age (70+ years) and three with missing data across multiple survey items, 348 participants were included. A total of 236 males (67.8%) and 112 (32.2%) women took part in the survey. Participants’ ages ranged from 27 to 69 years (mean age = 46.4 years (s.d. 9.61)); 257 (73.9%) participants were consultants, 89 (25.6%) were non-consultant grades and 2 (0.01%) did not disclose their grade. The number of consultants who were female was 66 (25.7%).

The three main specialties were general surgery, urology, and trauma and orthopaedic surgery. The full list of specialties, grades and place of work can be found in *Supplementary materials Table S1*.

*Table 1* displays the means, standard deviations and scale Cronbach alpha (α, a measure of reliability) for the sample on all the measures included in the models. All scales showed good-excellent internal consistency in the sample as indicated by α ranging from 0.763 to 0.917, suggesting that the scales were reliable measures.

**Table 1:** Descriptive data for surgeon sample on all scale measures of mental health, resilience, PF and personality

Measure	Maximum scale score	Score sample mean(s.d.)	Scale internal consistency α
DASS depression	42	6.79(7.39)	0.874
DASS anxiety	42	3.38(4.49)	0.763
DASS stress	42	10.94(7.55)	0.852
CBI work burnout	100	43.96(19.07)	0.864
CBI client burnout	100	32.88(19.76)	0.846
CBI personal burnout	100	48.80(17.42)	0.839
CBI total burnout	100	41.99(17.02)	0.917
BRS resilience	5	3.25(0.84)	0.881
WAAQ PF	49	33.66(6.64)	0.880
BFI neuroticism	5	2.62(0.82)	0.844
BFI extraversion	5	3.35(0.88)	0.875
BFI conscientiousness	5	4.21(0.54)	0.774

DASS, Depression Anxiety and Stress Scale (total score)^48^; CBI, Copenhagen Burnout Inventory (total score)^46^; BRS, Brief Resilience Scale (mean score)^50^; WAAQ, Work-Related Acceptance and Action Questionnaire (total score)^45^; BFI, Big Five Inventory—neuroticism, extraversion and conscientiousness subscales (mean scores)^43^; PF, psychological flexibility.

### Extraversion and mental health – testing PF and resilience as mediators

**Fig. 2:**
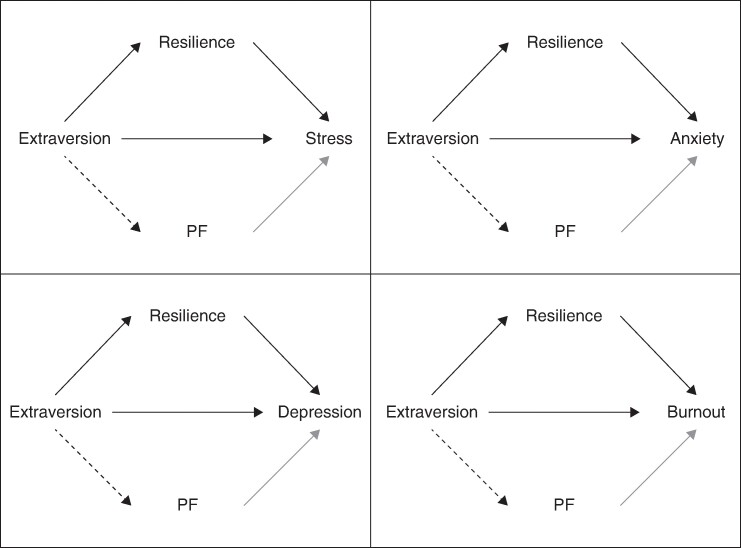
Diagram illustrating mediating pathways of resilience and psychological flexibility (PF) in the relationship between extraversion and each mental health measure (stress, anxiety, depression and burnout)

The mediation models examining extraversion (X) found that resilience (M1) mediated the relationships between extraversion and stress (b = −0.63, 95% c.i. −1.05 to −0.27), extraversion and anxiety (b = −0.30, 95% c.i. −0.52 to −0.13), extraversion and depression (b = −0.48, 95% c.i. −0.83 to −0.20), and extraversion and burnout (b = −1.39, 95% c.i. −2.29 to −0.65). These relationships had small or small-medium sized effects (*Fig. 2*).

Statistically significant pathways are represented as solid black lines, with dotted lines representing pathways that were not significant and grey lines representing significant relationships that do not form part of a mediated pathway. These models indicate that while there is a direct relationship between extraversion and all mental health outcomes, there is also a pathway mediated by resilience. PF did not mediate the relationship between extraversion and mental health; although there was a significant relationship between PF and all mental health outcomes, there was not a significant relationship between extraversion and PF.

However, within these models, PF (M2) did not mediate the relationships between extraversion and any mental health measure (*Supplementary materials, Tables S2*–*S4*).

### Conscientiousness and mental health – testing PF and resilience as mediators

The mediation models examining conscientiousness (X) found that resilience (M1) mediated the relationships between conscientiousness and stress (b = −0.74, 95% c.i. −1.40 to −0.17), conscientiousness and anxiety (b = −0.35, 95% c.i. −0.70 to −0.08), conscientiousness and depression (b = −0.64, 95% c.i. −1.24 to −0.13), and conscientiousness and burnout (b = −1.67, 95% c.i. −3.14 to −0.38). These relationships had small-medium effect sizes.

**Fig. 3:**
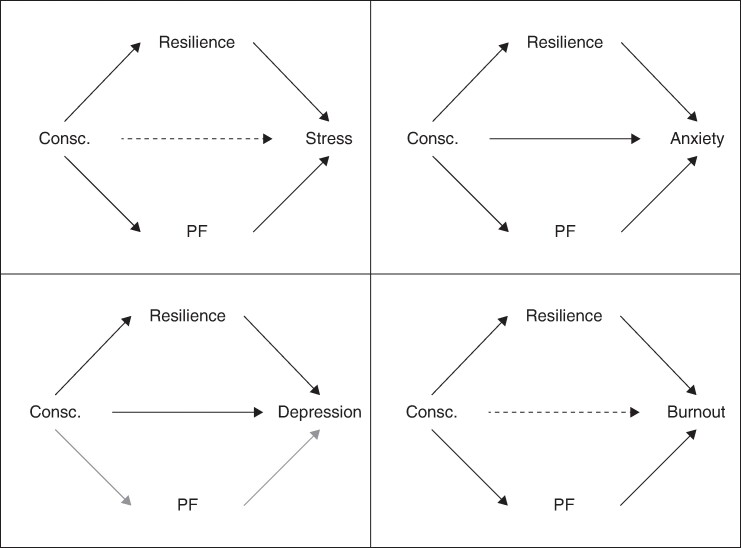
**Diagram illustrating mediating pathways of resilience and psychological flexibility (PF) in the relationship between conscientiousness (Consc**.) **and each mental health measure (stress, anxiety, depression and burnout)**

Within these models, PF (M2) mediated the relationships between conscientiousness and stress (b = −0.55, 95% c.i. −1.10 to −0.10), conscientiousness and anxiety (b = −0.30, 95% c.i. −0.63 to −0.03), and conscientiousness and burnout (b = −1.73, 95% c.i. −3.07 to −0.66). These relationships had small-medium effect sizes. However, PF did not mediate the relationship between conscientiousness and depression (see *Fig. 3*).

Statistically significant mediated pathways are represented as solid black lines, with dotted lines representing pathways that were not significant, and grey lines representing significant relationship where the mediated pathways was not significant overall. These models indicate that resilience mediated the relationship between conscientiousness and all mental health outcomes. PF mediated the relationship between conscientiousness and stress, conscientiousness and anxiety, and conscientiousness and burnout. While all individual relationships were significant in the conscientiousness-depression model, PF was not found to be a significant mediator within this model overall.

### Neuroticism and mental health – testing PF and resilience as mediators

The mediation models examining neuroticism (X) found that resilience (M1) did not mediate the relationship between neuroticism and stress, anxiety or depression (see *Table S3*). However, resilience did mediate the relationship between neuroticism and burnout (b = 2.48, 95% c.i. 0.97 to 4.00), with a medium-large sized effect.

**Fig. 4:**
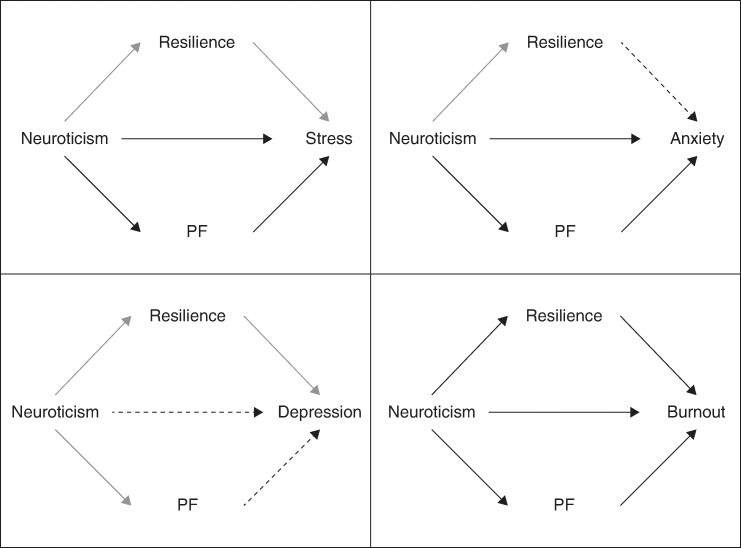
Diagram illustrating mediating pathways of resilience and psychological flexibility (PF) in the relationship between neuroticism and each mental health measure (stress, anxiety, depression and burnout)

Within these models, PF (M2) mediated the relationship between neuroticism and stress (b = 0.40, 95% c.i. 0.01 to 0.81), neuroticism and anxiety (b = 0.27, 95% c.i. 0.02 to 0.55), and neuroticism and burnout (b = 1.40, 95% c.i. 0.50 to 2.37). These relationships had small-medium effect sizes. PF did not, however, mediate the relationship between neuroticism and depression (see *Fig. 4*).

Statistically significant mediated pathways are represented as solid black lines, with dotted lines representing pathways that were not significant, and grey lines representing significant relationship where the mediated pathway was not significant overall. These models indicate that resilience mediated the relationship between neuroticism and burnout but did not mediate the relationship between neuroticism and any other mental health outcome. PF mediated the relationship between neuroticism and stress, neuroticism and anxiety, and neuroticism and burnout, but not between conscientiousness and depression.

## Discussion

This study highlights how two malleable psychological skills can impact mental health in surgeons. First, this work challenges the notion that surgeons should possess a particular personality type, by highlighting the mediating role of psychological skills in shaping the mental health and wellbeing of surgeons. Second, these findings suggest that enhancing resilience and PF is likely to be effective at reducing the mental health difficulties often associated with the challenges of surgical life. Given the stressors that surgeons face in their work, understanding how they can use malleable psychological skills to improve their mental health has obvious benefits for surgeons (for example better quality of life), their employers (for example reduced absenteeism) and potentially their patients (for example in better quality services). These results concur with findings in other populations^38,39^.

Interestingly, the authors found that resilience and PF played different roles depending on the personality factor explored. First, higher extraversion was linked with higher resilience, which in turn, explained lower stress, anxiety, depression and burnout. However, a similar relationship was not found for PF. Thus, the findings suggest that resilience is more important in explaining the extraversion-mental health link among surgeons.

In contrast, PF was more important than resilience in explaining the relationship between neuroticism and mental health; lower neuroticism was linked to higher PF, which was related to better mental health (less stress, anxiety and burnout). While this fits with previous research highlighting the importance of PF in explaining the link between neuroticism and mental health^38^, the authors are the first to explore this among surgeons and identify the differential contribution of PF and resilience.

Furthermore, the current findings indicated that both resilience and PF were significant mediators in the relationship between conscientiousness and better mental health; higher conscientiousness was linked to higher PF and resilience. In turn, both psychological factors explained the positive relationship between conscientiousness and stress, anxiety and burnout (with resilience additionally linked to lower depression). The previously reported positive link between conscientiousness and mental health^24,25^ may thus be partly explained by conscientious individuals possessing higher resilience and PF, which act to protect against mental health difficulties. Given that both these factors are malleable^33,56^, psychological interventions which target them (rather than a focus on personality per se) should be particularly useful.

These findings suggest that it is not merely the surgeon’s personality, but also their PF and resilience that influence their mental health. An obvious implication of these findings is that effort should be devoted to the development and testing of interventions that seek to enhance PF and resilience in surgeons. Moreover, given that it is possible that resilience and PF play slightly different roles depending on personality, ideally any interventions developed should address both resilience and PF.

Existing work recommends the importance of promoting interventions based on resilience skills in surgical settings^56,57^, with interventions based on mindfulness and cognitive-behavioural approaches shown to be effective^41^. Moreover, PF can be enhanced through mindfulness and acceptance-based practices, such as those developed from Acceptance and Commitment Therapy (ACT)^32^. ACT uses mindfulness and acceptance processes to build PF^32^. ACT-based interventions improve mental health outcomes for healthcare workers^58,59^, and the authors' RCT of a three-session ACT-based intervention shows promise with trainee surgeons^60^. Thus, these findings support the idea that developing psychological skills and qualities through psychological interventions might be particularly useful, regardless of personality ‘type’.

As these findings come from cross-sectional survey data, the implications and conclusions that are drawn about temporality of relationships are potentially limited. Future research would benefit from examining these mediated pathways longitudinally. Moreover, although data were collected from a moderate sample of surgeons (*n* = 348), this equates to 1.3% of the current number of surgeons in England alone (NHS Workforce Statistics^61^). The opportunistic nature of this sampling is likely limited by self-selection bias.

In addition, this sample may have over-represented women in surgery, given that around 32% of the overall sample were women, and around 25% of the consultants in the sample were female; this is a higher representation of women than is usually seen in surgical professions^62^. However, there is a great deal of variation in the number of women across surgical specialties, and a much higher number of women usually seen at lower grades^62^. It may be that the sample reflects a higher proportion of women early on in their consultant careers, which may also skew the sample. Furthermore, the survey data were collected before the coronavirus (COVID-19) pandemic. Given the impact of COVID-19 on surgical life^63^ it would be important to examine these relationships in surgeons following the pandemic.

Notably, in almost all of the authors' analyses, the mediators had small-medium effect sizes, explaining only some of the relationship between personality and wellbeing. This fits with the current understanding of mental health and burnout in healthcare settings^64,65^, where the relationship between personality, psychological skills and mental health is likely influenced by many factors including organizational and systemic issues. While the research highlights the important role of individual psychological skill in protecting against mental health difficulties in surgery, this does not diminish the vital role of organizations in prioritizing systemic interventions and structures that improve surgeon wellbeing and support more widely^66^.

## Supplementary Material

zrae060_Supplementary_Data

## Data Availability

Data is available on request.

## References

[1] Dimou FM, Eckelbarger D, Riall TS. Surgeon burnout: a systematic review. J Am Coll Surg 2016;222:1230–1239 27106639 10.1016/j.jamcollsurg.2016.03.022 PMC4884544

[2] Shanafelt TD, Gradishar WJ, Kosty M, Satele D, Chew H, Horn L et al Burnout and career satisfaction among US oncologists. J Clin Oncol 2014;32:678–686 24470006 10.1200/JCO.2013.51.8480 PMC3927737

[3] Oreskovich MR, Kaups KL, Balch CM, Hanks JB, Satele D, Sloan J et al Prevalence of alcohol use disorders among American surgeons. Arch Surg 2012;147:168–174 22351913 10.1001/archsurg.2011.1481

[4] Shanafelt TD, Balch CM, Dyrbye L, Bechamps G, Russell T, Satele D et al Special report: suicidal ideation among American surgeons. Arch Surg 2011;146:54–62 21242446 10.1001/archsurg.2010.292

[5] Grover R, Dua P, Juneja S, Chauhan L, Agarwal P, Khurana A. Depression, anxiety and stress in a cohort of registered practicing ophthalmic surgeons, post lockdown during COVID-19 pandemic in India. Ophthalmic Epidemiol 2021;28:322–329 33185487 10.1080/09286586.2020.1846757

[6] Lindeman B, Petrusa E, McKinley S, Hashimoto DA, Gee D, Smink DS et al Association of burnout with emotional intelligence and personality in surgical residents: can we predict who is most at risk? J Surg Educ 2017;74:e22–e30 29198973 10.1016/j.jsurg.2017.11.001

[7] Shanafelt TD, Balch CM, Bechamps GJ, Russell T, Dyrbye L, Satele D et al Burnout and career satisfaction among American surgeons. Ann Surg 2009;250:463–471 19730177 10.1097/SLA.0b013e3181ac4dfd

[8] Turner K, Bolderston H, Thomas K, Greville-Harris M, Withers C, McDougall S. Impact of adverse events on surgeons. Br J Surg 2022;109:308–310 35084452 10.1093/bjs/znab447

[9] Sauer-Zavala S, Barlow DH. Neuroticism: A New Framework for Emotional Disorders and Their Treatment. New York: The Guilford Press, 2021

[10] Stoeber J, Otto K, Dalbert C. Perfectionism and the big five: conscientiousness predicts longitudinal increases in self-oriented perfectionism. Pers Individ Dif 2009;47:363–368

[11] Mooradian TA, Swan KS. Personality-and-culture: the case of national extraversion and word-of-mouth. J Bus Res 2006;59:778–785

[12] Sier VQ, Schmitz RF, Schepers A, van der Vorst JR. Exploring the surgical personality. Surgeon 2023;21:1–7 35241372 10.1016/j.surge.2022.01.008

[13] Struijs SY, de Jong PJ, Jeronimus BF, van der Does W, Riese H, Spinhoven P. Psychological risk factors and the course of depression and anxiety disorders: a review of 15 years NESDA research. J Affect Disord 2021;295:1347–1359 34706448 10.1016/j.jad.2021.08.086

[14] Hayes N, Joseph S. Big 5 correlates of three measures of subjective well-being. Pers Individ Dif 2003;34:723–727

[15] Geuens N, Van Bogaert P, Franck E. Vulnerability to burnout within the nursing workforce – the role of personality and interpersonal behaviour. J Clin Nurs 2017;26:4622–4633 28295750 10.1111/jocn.13808

[16] Holman A, Gavrilescu I-M, Muraru I-D, Petrariu F. Alexithymia and the big five personality traits as predictors of burnout among medical students. Med Surg J 2018;122:592–602

[17] Baumgarten C, Michinov E, Rouxel G, Bonneterre V, Gay E, Roche PH. Personal and psychosocial factors of burnout: a survey within the French neurosurgical community. PLoS One 2020;15:e0233137 32469930 10.1371/journal.pone.0233137 PMC7259549

[18] Prins DJ, van Vendeloo SN, Brand PLP, Van der Velpen I, de Jong K, van den Heijkant F et al The relationship between burnout, personality traits, and medical specialty. A national study among Dutch residents. Med Teach 2019;41:584–590 30394166 10.1080/0142159X.2018.1514459

[19] Iorga M, Socolov V, Muraru D, Dirtu C, Soponaru C, Ilea C et al Factors influencing burnout syndrome in obstetrics and gynecology physicians. Biomed Res Int 2017;2017:9318534 29359161 10.1155/2017/9318534 PMC5735583

[20] Pereira-Lima K, Loureiro SR, Crippa JA. Mental health in medical residents: relationship with personal, work-related, and sociodemographic variables. Braz J Psychiatry 2016;38:318–324 27192216 10.1590/1516-4446-2015-1882 PMC7111348

[21] Fino E, Sun S. Let us create!: the mediating role of creative self-efficacy between personality and mental well-being in university students. Pers Individ Dif 2022;188:111444

[22] Anderson KW, McLean PD. Conscientiousness in depression: tendencies, predictive utility, and longitudinal stability. Cognit Ther Res 1997;21:223–238

[23] McManus IC, Keeling A, Paice E. Stress, burnout and doctors’ attitudes to work are determined by personality and learning style: a twelve year longitudinal study of UK medical graduates. BMC Med 2004;2:29 15317650 10.1186/1741-7015-2-29 PMC516448

[24] Rogers ME, Creed PA, Searle J. Person and environmental factors associated with well-being in medical students. Pers Individ Dif 2012;52:472–477

[25] Tyssen R, Dolatowski FC, Røvik JO, Thorkildsen RF, Ekeberg O, Hem E et al Personality traits and types predict medical school stress: a six-year longitudinal and nationwide study. Med Educ 2007;41:781–787 17661886 10.1111/j.1365-2923.2007.02802.x

[26] Rutter M . Resilience in the face of adversity. Protective factors and resistance to psychiatric disorder. Br J Psychiatry 1985;147:598–611 3830321 10.1192/bjp.147.6.598

[27] Southwick SM, Bonanno GA, Masten AS, Panter-Brick C, Yehuda R. Resilience definitions, theory, and challenges: interdisciplinary perspectives. Eur J Psychotraumatol 2014:5:25338 10.3402/ejpt.v5.25338 PMC4185134 25317257

[28] Walker C, Gleaves A, Grey J. Can students within higher education learn to be resilient and, educationally speaking, does it matter? Educ Stud 2006;32:251–264

[29] Rogers D . Which educational interventions improve healthcare professionals’ resilience? Med Teach 2016;38:1236–1241 27573430 10.1080/0142159X.2016.1210111

[30] Murden F, Bailey D, Mackenzie F, Oeppen RS, Brennan PA. The impact and effect of emotional resilience on performance: an overview for surgeons and other healthcare professionals. Br J Oral Maxillofac Surg 2018;56:786–790 30220608 10.1016/j.bjoms.2018.08.012

[31] Ortiz-Fune C, Kanter J, Arias M. Burnout in mental health professionals: the roles of psychological flexibility, awareness, courage, and love. Clín Salud 2020;31:85–90

[32] Hayes SC, Luoma JB, Bond FW, Masuda A, Lillis J. Acceptance and commitment therapy: model, processes and outcomes. Behav Res Ther 2006;44:1–25 16300724 10.1016/j.brat.2005.06.006

[33] Gloster AT, Meyer AH, Lieb R. Psychological flexibility as a malleable public health target: evidence from a representative sample. J Contextual Behav Sci 2017;6:166–171

[34] Kashdan TB, Rottenberg J. Psychological flexibility as a fundamental aspect of health. Clin Psychol Rev 2010;30:865–878 21151705 10.1016/j.cpr.2010.03.001 PMC2998793

[35] Garner EV, Golijani-Moghaddam N. Relationship between psychological flexibility and work-related quality of life for healthcare professionals: a systematic review and meta-analysis. J Contextual Behav Sci 2021;21:98–112

[36] Yao Y, Jing X, Lu L. Interaction of job-related psychological flexibility, coping style and personality types in depression in Chinese physicians: a cross-section study. Medicine (Baltimore) 2022;101:e30838 36181024 10.1097/MD.0000000000030838 PMC9524922

[37] Johns G, Waddington L, Samuel V. Prevalence and predictors of mental health outcomes in UK doctors and final year medical students during the COVID-19 pandemic. J Affect Disord 2022;311:267–275 35569608 10.1016/j.jad.2022.05.024 PMC9098653

[38] Steenhaut P, Rossi G, Demeyer I, De Raedt R. How is personality related to well-being in older and younger adults? The role of psychological flexibility. Int Psychogeriatr 2019;31:1355–1365 30547852 10.1017/S1041610218001904

[39] Shi M, Liu L, Wang ZY, Wang L. The mediating role of resilience in the relationship between big five personality and anxiety among Chinese medical students: a cross-sectional study. PLoS One 2015;10:e0119916 25794003 10.1371/journal.pone.0119916 PMC4368674

[40] Flaxman PE, Bond FW. A randomised worksite comparison of acceptance and commitment therapy and stress inoculation training. Behav Res Ther 2010;48:816–820 20627269 10.1016/j.brat.2010.05.004

[41] Joyce S, Shand F, Tighe J, Laurent SJ, Bryant RA, Harvey SB. Road to resilience: a systematic review and meta-analysis of resilience training programmes and interventions. BMJ Open 2018;8:e017858 10.1136/bmjopen-2017-017858 PMC6009510 29903782

[42] Qualtrics XM . Make Every Interaction an Experience That Matters. London: Qualtrics XM, 2024. https://www.qualtrics.com/uk (updated 2024; accessed 9 May 2024)

[43] Fritz MS, MacKinnon DP. Required sample size to detect the mediated effect. Psychol Sci 2007;18:233–239 17444920 10.1111/j.1467-9280.2007.01882.x PMC2843527

[44] John OP, Srivastava S. The big five trait taxonomy: history, measurement, and theoretical perspectives. In: Pervin LA, John OP (eds.), *Handbook of Personality: Theory and Research* (2nd edn). New York: The Guilford Press, 1999, 102–138

[45] Schmitt DP, Allik J, McCrae RR, Benet-Martínez V. The geographic distribution of big five personality traits: patterns and profiles of human self-description across 56 nations. J Cross Cult Psychol 2007;38:173–212

[46] Bond FW, Lloyd J, Guenole N. The work-related acceptance and action questionnaire: initial psychometric findings and their implications for measuring psychological flexibility in specific contexts. J Occup Organ Psychol 2013;86:331–347

[47] Smith BW, Dalen J, Wiggins K, Tooley E, Christopher P, Bernard J. The brief resilience scale: assessing the ability to bounce back. Int J Behav Med 2008;15:194–200 18696313 10.1080/10705500802222972

[48] Windle G, Bennett KM, Noyes J. A methodological review of resilience measurement scales. Health Qual Life Outcomes 2011;9:8 21294858 10.1186/1477-7525-9-8 PMC3042897

[49] Lovibond SH, Lovibond PF. Manual for the Depression Anxiety Stress Scales (2nd edn). Sydney: Psychology Foundation of Australia, 1995

[50] Bibi A, Lin M, Zhang XC, Margraf J. Psychometric properties and measurement invariance of depression, anxiety and stress scales (DASS-21) across cultures. Int J Psychol 2020;55:916–925 32253755 10.1002/ijop.12671

[51] Kristensen TS, Borritz M, Villadsen E, Christensen KB. The Copenhagen Burnout Inventory: a new tool for the assessment of burnout. Work Stress 2005;19:192–207

[52] Thrush CR, Gathright MM, Atkinson T, Messias EL, Guise JB. Psychometric properties of the Copenhagen Burnout Inventory in an academic healthcare institution sample in the U.S. Eval Health Prof 2021;44:400–405 32539552 10.1177/0163278720934165

[53] Caesar B, Barakat A, Bernard C, Butler D. Evaluation of physician burnout at a major trauma centre using the Copenhagen Burnout Inventory: cross-sectional observational study. Ir J Med Sci 2020;189:1451–1456 32285375 10.1007/s11845-020-02223-5

[54] Lin TH . A comparison of multiple imputation with EM algorithm and MCMC method for quality of life missing data. Qual Quant 2010;44:277–287

[55] Hayes AF. Introduction to Mediation, Moderation, and Conditional Process Analysis: A Regression-Based Approach. 3rd ed. New York: Guilford Press, 2022, 732

[56] Naviaux AF, Barbier L, Chopinet S, Janne P, Gourdin M. Ways of preventing surgeon burnout. J Visc Surg 2023;160:33–38 36257890 10.1016/j.jviscsurg.2022.09.005

[57] Mahmoud NN, Rothenberger D. From burnout to well-being: a focus on resilience. Clin Colon Rectal Surg 2019;32:415–423 31686993 10.1055/s-0039-1692710 PMC6824889

[58] Waters CS, Frude N, Flaxman PE, Boyd J. Acceptance and commitment therapy (ACT) for clinically distressed health care workers: waitlist-controlled evaluation of an ACT workshop in a routine practice setting. Br J Clin Psychol 2018;57:82–98 28857254 10.1111/bjc.12155

[59] Prudenzi A, Graham CD, Flaxman PE, Wilding S, Day F, O’Connor DB. A workplace acceptance and commitment therapy (ACT) intervention for improving healthcare staff psychological distress: a randomised controlled trial. PLoS One 2022;17:e0266357 35442963 10.1371/journal.pone.0266357 PMC9020690

[60] Richer S, Bolderston H, Turner K, McDougall S, Thomas K. ACT based resilience training for surgeons. In: *Association for Contextual Behavioral Science World Conference; 25–30 June 2019; Dublin, Ireland*

[61] NHS England . *NHS Workforce Statistics - August 2023 (Including Selected Provisional Statistics for September 2023)* [Internet]. England: NHS England, 2023. https://digital.nhs.uk/data-and-information/publications/statistical/nhs-workforce-statistics/august-2023 (updated November 2023; accessed 9 May 2024)

[62] Moberly T . A fifth of surgeons in England are female. BMJ 2018;363:k4530 30377150 10.1136/bmj.k4530

[63] Tan YQ, Wang Z, Yap QV, Chan YH, Ho RC, Hamid A et al Psychological health of surgeons in a time of COVID-19: a global survey. Ann Surg 2023;277:50–56 33491983 10.1097/SLA.0000000000004775 PMC9762613

[64] Galaiya R, Kinross J, Arulampalam T. Factors associated with burnout syndrome in surgeons: a systematic review. Ann R Coll Surg Engl 2020;102:401–407 32326734 10.1308/rcsann.2020.0040 PMC7388944

[65] Teoh K, Singh J, Medisauskaite A, Hassard J. Doctors’ perceived working conditions, psychological health and patient care: a meta-analysis of longitudinal studies. Occup Environ Med 2023;80:61–69 36635099 10.1136/oemed-2022-108486

[66] Hirayama M, Fernando S. Burnout in surgeons and organisational interventions. J Royal Soc Med 2016;109:400–403

