# Supplementary material for: Association of resilience and psychological flexibility with surgeons’ mental wellbeing
Source: BJS Open. 2024 Jul 23;8(4):zrae060. doi: 10.1093/bjsopen/zrae060 (PMC11264141; doi:10.1093/bjsopen/zrae060)
Supplement: zrae060_Supplementary_Data [file zrae060_supplementary_data.docx]

**Association of resilience and psychological flexibility with surgeons’ mental wellbeing**

Maddy Greville-Harris^1^, Catherine Withers^1^, Agata Wezyk^1^, Kevin Thomas^1^, Helen Bolderston^1^, Amy Kane^1^, Sine McDougall^1^ & Kevin J. Turner^1,2^

^1^Department of Psychology, Poole House, Bournemouth University, Fern Barrow, Poole, BH12 5BB.

^2^Department of Urology, University Hospitals Dorset, Castle Lane East, Bournemouth. BH7 7DW.

**Corresponding author:** Kevin Turner, University Hospitals Dorset, Royal Bournemouth and Christchurch Hospitals NHS Foundation Trust, Castle Lane East, Bournemouth BH7 7DW, UK. e-mail: [kevin.turner@uhd.nhs.uk](mailto:kevin.turner@uhd.nhs.uk); **ORCID ID: 0009-0009-9177-3917; Twitter: @urodoco**

| **Supplementary Appendices** |  |
| --- | --- |
| Measure A1: Work-Related Acceptance and Action Questionnaire (WAAQ) | *Page 2* |
| Measure A2: Brief Resilience Scale (BRS) | *Page 3* |
| Measure A3: Copenhagen Burnout Inventory (CBI) | *Page 4* |
| Measure A4: Depression, Anxiety and Stress Scale (DASS) | *Page 5* |
| Measure A5: Big Five Inventory (BFI) | *Page 6* |
| **Supplementary Figures and Tables** |  |
| Table S1: Count and frequency of surgeons in each specialty in our sample | *Page 8* |
| Table S2: Summary of model statistics for mediation models with Extraversion as the predictor variable [X] | *Page 9* |
| Table S3: Summary of model statistics for mediation models with Conscientiousness as the predictor variable [X] | *Page 10* |
| Table S4: Summary of model statistics for mediation models with Neuroticism as the predictor variable [X] | *Page 11* |
|  |  |

**Supplementary Appendices**

**Measure A1: Work-Related Acceptance and Action Questionnaire (WAAQ)**

|  | Never true | | Very Seldom True | | Seldom True | | Sometimes True | | Frequently true | | Almost always true | | Always True | |  |
| --- | --- | --- | --- | --- | --- | --- | --- | --- | --- | --- | --- | --- | --- | --- | --- |
| I am able to work effectively in spite of any personal worries that I have | |  | |  | |  | |  | |  | |  | |  | |
| I can admit to my mistakes at work and still be successful | |  | |  | |  | |  | |  | |  | |  | |
| I can still work very effectively, even if I am nervous about something | |  | |  | |  | |  | |  | |  | |  | |
| Worries do not get in the way of my success | |  | |  | |  | |  | |  | |  | |  | |
| I can perform as required no matter how I feel | |  | |  | |  | |  | |  | |  | |  | |
| I can work effectively, even when I doubt myself | |  | |  | |  | |  | |  | |  | |  | |
| My thoughts and feelings do not get in the way of my work | |  | |  | |  | |  | |  | |  | |  | |

**Measure A2: Brief Resilience Scale (BRS)**

|  | Strongly disagree | Disagree | Neutral | Agree | Strongly agree |
| --- | --- | --- | --- | --- | --- |
| I tend to bounce back quickly after hard times |  |  |  |  |  |
| I have a hard time making it through stressful events |  |  |  |  |  |
| It does not take me long to recover from a stressful event |  |  |  |  |  |
| It is hard for me to snap back when something bad happens |  |  |  |  |  |
| I usually come through difficult times with little trouble |  |  |  |  |  |
| I tend to take a long time to get over set-backs in my life |  |  |  |  |  |

**Measure A4: Copenhagen Burnout Inventory (CBI)**

|  | | Always | | Often | | Sometimes | | Seldom | | Never |
| --- | --- | --- | --- | --- | --- | --- | --- | --- | --- | --- |
| How often do you feel tired? | |  | |  | |  | |  | |  |
| Do you feel worn out at the end of the working day? | |  | |  | |  | |  | |  |
| Are you tired of working with patients? | |  | |  | |  | |  | |  |
| How often do you feel worn out? | |  | |  | |  | |  | |  |
| Are you exhausted in the morning at the thought of another day at work? | |  | |  | |  | |  | |  |
| How often are you physically exhausted? | |  | |  | |  | |  | |  |
| Do you have enough energy for family and friends during leisure time? | |  | |  | |  | |  | |  |
| Do you sometimes wonder how long you will be able to continue working with patients? | |  | |  | |  | |  | |  |
| How often are you emotionally exhausted? | |  | |  | |  | |  | |  |
| How often do you feel weak and susceptible to illness? | |  | |  | |  | |  | |  |
| How often do you think 'I can't take it anymore'? | |  | |  | |  | |  | |  |
| Do you feel that every working hour is tiring for you? | |  | |  | |  | |  | |  |
|  | To a very high degree | | To a high degree | | Somewhat | | To a low degree | | To a very low degree | |
| Does it drain your energy to work with patients? |  | |  | |  | |  | |  | |
| Is your work emotionally exhausting? |  | |  | |  | |  | |  | |
| Do you find it frustrating to work with patients? |  | |  | |  | |  | |  | |
| Do you find it hard to work with patients? |  | |  | |  | |  | |  | |
| Do you feel that you give more than you get back when you work with patients? |  | |  | |  | |  | |  | |
| Do you feel burnt out because of your work? |  | |  | |  | |  | |  | |
| Does your work frustrate you? |  | |  | |  | |  | |  | |

**Measure A5: Depression, Anxiety and Stress Scale (DASS)**

|  | Did not apply to me at all | Applied to me to some degree, or some of the time | Applied to me to a considerable degree, or a good part of the time | Applied to me very much, or most of the time |
| --- | --- | --- | --- | --- |
| I found it hard to wind down |  |  |  |  |
| I was aware of dryness of my mouth |  |  |  |  |
| I couldn't seem to experience any positive feeling at all |  |  |  |  |
| I experienced breathing difficulty (eg, excessively rapid breathing, breathlessness in the absence of physical exertion) |  |  |  |  |
| I found it difficult to work up the initiative to do things |  |  |  |  |
| I tended to over-react to situations |  |  |  |  |
| I experienced trembling (e.g., in the hands) |  |  |  |  |
| I felt that I was using a lot of nervous energy |  |  |  |  |
| I was worried about situations in which I might panic and make a fool of myself |  |  |  |  |
| I felt that I had nothing to look forward to |  |  |  |  |
| I found myself getting agitated |  |  |  |  |
| I found it difficult to relax |  |  |  |  |
| I felt down-hearted and blue |  |  |  |  |
| I was intolerant of anything that kept me from getting on with what I was doing |  |  |  |  |
| I felt I was close to panic |  |  |  |  |
| I was unable to become enthusiastic about anything |  |  |  |  |
| I felt I wasn't worth much as a person |  |  |  |  |
| I felt that I was rather touchy |  |  |  |  |
| I was aware of the action of my heart in the absence of physical exertion (eg, sense of heart rate increase, heart missing a beat) |  |  |  |  |
| I felt scared without any good reason |  |  |  |  |
| I felt that life was meaningless |  |  |  |  |

**Measure A6: Big Five Inventory (BFI)**

I see myself as someone who…

|  | Disagree Strongly | Disagree a little | Neither agree nor disagree | Agree a little | Agree Strongly |
| --- | --- | --- | --- | --- | --- |
| Is talkative |  |  |  |  |  |
| Tends to find fault with others |  |  |  |  |  |
| Does a thorough job |  |  |  |  |  |
| Is depressed/blue |  |  |  |  |  |
| Is original/comes up with new ideas |  |  |  |  |  |
| Is reserved |  |  |  |  |  |
| Is helpful and unselfish with others |  |  |  |  |  |
| Can be somewhat careless |  |  |  |  |  |
| Is relaxed, handles stress well |  |  |  |  |  |
| Is curious about many different things |  |  |  |  |  |
| Is full of energy |  |  |  |  |  |
| Starts quarrels with others |  |  |  |  |  |
| Is a reliable worker |  |  |  |  |  |
| Can be tense |  |  |  |  |  |
| Is ingenious, a deep thinker |  |  |  |  |  |
| Generates a lot of enthusiasm |  |  |  |  |  |
| Has a forgiving nature |  |  |  |  |  |
| Tends to be disorganised |  |  |  |  |  |
| Worries a lot |  |  |  |  |  |
| Has an active imagination |  |  |  |  |  |
| Tends to be quiet |  |  |  |  |  |
|  |  |  |  |  |  |

|  | Disagree Strongly | Disagree a little | Neither agree nor disagree | Agree a little | Agree Strongly |
| --- | --- | --- | --- | --- | --- |
| Tends to be lazy |  |  |  |  |  |
| Is emotionally stable, not easily upset |  |  |  |  |  |
| Is inventive |  |  |  |  |  |
| Has an assertive personality |  |  |  |  |  |
| Can be cold and aloof |  |  |  |  |  |
| Perseveres until the task is finished |  |  |  |  |  |
| Can be moody |  |  |  |  |  |
| Values artistic, aesthetic experiences |  |  |  |  |  |
| Is sometimes shy, inhibited |  |  |  |  |  |
| Is considerate and kind to almost everyone |  |  |  |  |  |
| Does things efficiently |  |  |  |  |  |
| Remains calm in tense situations |  |  |  |  |  |
| Is a reliable worker |  |  |  |  |  |
| Prefers work that is routine |  |  |  |  |  |
| Is outgoing, sociable |  |  |  |  |  |
| Is sometimes rude to others |  |  |  |  |  |
| Makes plans and follows through with them |  |  |  |  |  |
| Gets nervous easily |  |  |  |  |  |
| Likes to reflect, play with ideas |  |  |  |  |  |
| Has few artistic interests |  |  |  |  |  |
| Likes to cooperate with others |  |  |  |  |  |
| Is easily distracted |  |  |  |  |  |
| Is sophisticated in art, music, or literature |  |  |  |  |  |
| Is generally trusting |  |  |  |  |  |

**Supplementary Tables**

Table S1.

*Count and frequency of surgeons in each place of work, grade and specialty in our sample*

| Workplace and current grade category | Count (%) |
| --- | --- |
| Principal Place of Work (n=348): |  |
| District General Hospital | 145 (41.7) |
| University Teaching Hospital | 190 (54.6) |
| Private Practice | 13 (3.7) |
| Current Grade (n=346): |  |
| Consultant | 257 (73.9) |
| StR8 | 2 (0.6) |
| StR7 | 25 (7.2) |
| StR6 | 11 (3.2) |
| StR5 | 14 (4.0) |
| StR4 | 3 (1.4) |
| StR3 | 14 (4.0) |
| Staff grade/associate specialist | 18 (5.2) |
| Specialty (n=348) |  |
| General surgery | 81 (23.3) |
| Urology | 75 (21.6) |
| Trauma and Orthopaedic surgery | 51 (14.7) |
| Paediatric surgery | 27 (7.8) |
| Plastic surgery | 18 (5.2) |
| Vascular surgery | 16 (4.6) |
| Obstetrics/gynaecology | 15 (4.3) |
| Oral/Dental and maxillofacial surgery | 14 (4.0) |
| Otolaryngology | 13 (3.7) |
| Ophthalmology | 13 (3.7) |
| Colorectal surgery | 7 (2.0) |
| Breast surgery | 5 (1.4) |
| Cardiothoracic surgery | 4 (1.1) |
| Academic surgery | 4 (1.1) |
| Neurosurgery | 3 (0.9) |
| Other | 2 (0.6) |
|  |  |

Table S2.

*Summary of model statistics for mediation models with Extraversion as the predictor variable [X]*

| Outcome variable [Y] | Mediators [M1, M2] | Mediator effects | | | | | Path A | | | | Path B | | Path C | | | | Path C’ | |
| --- | --- | --- | --- | --- | --- | --- | --- | --- | --- | --- | --- | --- | --- | --- | --- | --- | --- | --- |
|  |  | *b* | *LLCI* | *ULCI* | | *IE^#^* | R^2^ | F | *b* | SE | *b* | SE | R^2^ | F | *b* | SE | *b* | SE |
| Model 1: Stress | Resilience | **-0.63** | **-1.05** | **-0.27** | **-.073** | | .05 | 16.8 | 0.21** | .050 | -3.05** | .518 | .03 | 9.7 | -1.41** | .455 | -0.68 | .419 |
|  | Psychological Flexibility | -0.10 | -0.31 | 0.04 | -0.012 | | .01 | 2.00 | 0.57 | .405 | -0.18** | .064 |  |  |  |  |  |  |
| Model 2: Anxiety | Resilience | **-0.30** | **-0.52** | **-0.13** | **-0.060** | | .05 | 16.8  2.00 | 0.21** | .050 | -1.48** | .320 | .01 | 4.8 | -0.60** | .272 | -0.23 | .258 |
|  | Psychological Flexibility | -0.06 | -0.18 | 0.03 | -0.012 | | .01 |  | 0.57 | .405 | -0.11** | .040 |  |  |  |  |  |  |
| Model 3: Depression | Resilience | **-0.48** | **-0.83** | **-0.20** | **-0.058** | | .05 | 16.8  2.00 | 0.21** | .050 | -2.36** | .511 | .07 | 27.3 | -2.27** | .434 | -1.69** | .413 |
|  | Psychological Flexibility | -0.10 | -0.30 | 0.05 | -0.012 | | .01 |  | 0.57 | .405 | -0.17** | .063 |  |  |  |  |  |  |
| Model 4: Burnout | Resilience | **-1.39** | **-2.29** | **-0.65** | **-0.072** | | .05 | 16.8  2.00 | 0.21** | .050 | -6.75** | 1.14 | .04 | 13.2 | -3.70** | 1.02 | -2.01** | .921 |
|  | Psychological Flexibility | -0.31 | -0.86 | 0.14 | -0.016 | | .01 |  | 0.57 | .405 | -0.54** | .141 |  |  |  |  |  |  |

*Note:* Significant mediator effects in bold; IE^#^ is the standardised indirect effect (small effect≥ .01, medium effect ≥ .09, large effect ≥ .25); **= significant p<.01; * significant p<.05

Table S3.

*Summary of model statistics for mediation models with Conscientiousness as the predictor variable [X]*

| Outcome variable [Y] | Mediators [M1, M2] | Mediator effects | | | | Path A | | | | Path B | | Path C | | | | Path C’ | |
| --- | --- | --- | --- | --- | --- | --- | --- | --- | --- | --- | --- | --- | --- | --- | --- | --- | --- |
|  |  | *b* | *LLCI* | *ULCI* | *IE^#^* | R^2^ | F | *b* | SE | *b* | SE | R^2^ | F | *b* | SE | *b* | SE |
| Model 1: Stress | Resilience | **-0.74** | **-1.40** | **-0.17** | **-0.053** | .02 | 7.6 | 0.23** | .083 | -3.22** | .510 | .01 | 3.8 | -1.47 | .750 | -0.18 | .695 |
|  | Psychological Flexibility | **-0.55** | **-1.10** | **-0.10** | **-0.039** | .07 | 24.3 | 3.16** | .642 | -0.17** | .066 |  |  |  |  |  |  |
| Model 2: Anxiety | Resilience | **-0.35** | **-0.70** | **-0.08** | **-0.042** | .02 | 7.6 | 0.23** | .083 | -1.53** | .312 | .03 | 10.2 | -1.41** | .442 | -0.76 | .425 |
|  | Psychological Flexibility | **-0.30** | **-0.63** | **-0.03** | **-0.036** | .07 | 24.3 | 3.16** | .642 | -0.09* | .040 |  |  |  |  |  |  |
| Model 3: Depression | Resilience | **-0.64** | **-1.24** | **-0.13** | **-0.047** | .02 | 7.6 | 0.23** | .083 | -2.78* | .508 | .03 | 10.9 | -2.40** | .726 | -1.34 | .693 |
|  | Psychological Flexibility | -0.41 | -0.95 | 0.02 | -0.030 | .07 | 24.3 | 3.16** | .642 | -0.13* | .066 |  |  |  |  |  |  |
| Model 4: Burnout | Resilience | **-1.67** | **-3.14** | **-0.38** | **0.053** | .02 | 7.6 | 0.23** | .083 | -7.27** | 1.12 | .01 | 1.7 | -2.24 | 1.70 | 1.16 | 1.53 |
|  | Psychological Flexibility | **-1.73** | **-3.07** | **-0.66** | **0.055** | .07 | 24.3 | 3.16** | .642 | -0.55** | .145 |  |  |  |  |  |  |

*Note:* Significant mediator effects in bold; IE^#^ is the standardised indirect effect (small effect≥ .01, medium effect ≥ .09, large effect ≥ .25); **= significant p<.01; * significant p<.05

Table S4.

*Summary of model statistics for mediation models with Neuroticism as the predictor variable [X]*

| Outcome variable [Y] | Mediators [M1, M2] | Mediator effects | | | | Path A | | | | Path B | | | Path C | | | | Path C’ | |
| --- | --- | --- | --- | --- | --- | --- | --- | --- | --- | --- | --- | --- | --- | --- | --- | --- | --- | --- |
|  |  | *b* | *LLCI* | *ULCI* | *IE^#^* | R^2^ | F | *b* | SE | *b* | | SE | R^2^ | F | *b* | SE | *b* | SE |
| Model 1: Stress | Resilience | 0.71 | -0.15 | 1.51 | 0.078 | .35 | 184.9 | -0.60** | .045 | -1.19* | .540 | | .30 | 145.5 | 5.01** | .416 | 3.89** | .508 |
|  | Psychological Flexibility | **0.40** | **0.01** | **0.81** | **0.044** | .15 | 61.7 | -3.15** | .402 | -0.13* | .060 | |  |  |  |  |  |  |
| Model 2: Anxiety | Resilience | 0.39 | -0.06 | 0.83 | 0.072 | .35 | 184.9 | -0.60** | .045 | -0.65 | .346 | | .19 | 79.5 | 2.37** | .266 | 1.70** | .325 |
|  | Psychological Flexibility | **0.27** | **0.02** | **0.55** | **0.050** | .15 | 61.7 | -3.15** | .402 | -0.09* | .038 | |  |  |  |  |  |  |
| Model 3: Depression | Resilience | 0.70 | -0.03 | 1.37 | 0.077 | .35 | 184.9 | -0.60** | .045 | -1.15* | .559 | | .22 | 96.4 | 4.21 | .429 | 3.13 | .525 |
|  | Psychological Flexibility | 0.38 | -0.02 | 0.79 | 0.042 | .15 | 61.7 | -3.15** | .402 | -0.12 | .062 | |  |  |  |  |  |  |
| Model 4: Burnout | Resilience | **2.48** | **0.97** | **4.00** | **0.12** | .35 | 184.9 | -0.60** | .045 | -4.09** | 1.24 | | .23 | 103.0 | 9.95** | .980 | 6.07** | 1.17 |
|  | Psychological Flexibility | **1.40** | **0.50** | **2.37** | **.070** | .15 | 61.7 | -3.15** | .402 | 0.44** | .137 | |  |  |  |  |  |  |

*Note:* Significant mediator effects in bold; IE^#^ is the standardised indirect effect (small effect≥ .01, medium effect ≥ .09, large effect ≥ .25); **= significant p<.01; * significant p<.05
